# Metallic Engineered Nanomaterials and Ocular Toxicity: A Current Perspective

**DOI:** 10.3390/pharmaceutics14050981

**Published:** 2022-05-03

**Authors:** Krista M. Cosert, Soohyun Kim, Iman Jalilian, Maggie Chang, Brooke L. Gates, Kent E. Pinkerton, Laura S. Van Winkle, Vijay Krishna Raghunathan, Brian C. Leonard, Sara M. Thomasy

**Affiliations:** 1 Department of Surgical and Radiological Sciences, School of Veterinary Medicine, University of California Davis, Davis, CA 95616, USA; kcosert@ucdavis.edu (K.M.C.); shvkim@ucdavis.edu (S.K.); iman.jalilian@yale.edu (I.J.); magchang@ucdavis.edu (M.C.); blgates@ucdavis.edu (B.L.G.); bcleonard@ucdavis.edu (B.C.L.); 2 Center for Health and the Environment, University of California Davis, Davis, CA 95616, USA; kepinkerton@ucdavis.edu (K.E.P.); lsvanwinkle@ucdavis.edu (L.S.V.W.); 3 Department of Anatomy, Physiology and Cell Biology, School of Veterinary Medicine, University of California Davis, Davis, CA 95616, USA; 4 Department of Basic Sciences, College of Optometry, University of Houston, Houston, TX 77004, USA; vraghuna@central.uh.edu; 5 The Ocular Surface Institute, College of Optometry, University of Houston, Houston, TX 77004, USA; 6 Department of Biomedical Engineering, Cullen College of Engineering, University of Houston, Houston, TX 77204, USA; 7 Department of Ophthalmology & Vision Science, School of Medicine, University of California Davis, Davis, CA 95616, USA

**Keywords:** metallic engineered nanomaterials, ocular toxicity, eye, metallic nanoparticles, corneal wound healing

## Abstract

The ocular surface, comprised of the transparent cornea, conjunctiva, and protective tear film, forms a protective barrier defending deeper structures of the eye from particulate matter and mechanical trauma. This barrier is routinely exposed to a multitude of naturally occurring and engineered nanomaterials (ENM). Metallic ENMs are particularly ubiquitous in commercial products with a high risk of ocular exposure, such as cosmetics and sunscreens. Additionally, there are several therapeutic uses for metallic ENMs owing to their attractive magnetic, antimicrobial, and functionalization properties. The increasing commercial and therapeutic applications of metallic ENMs come with a high risk of ocular exposure with poorly understood consequences to the health of the eye. While the toxicity of metallic ENMs exposure has been rigorously studied in other tissues and organs, further studies are necessary to understand the potential for adverse effects and inform product usage for individuals whose ocular health may be compromised by injury, disease, or surgical intervention. This review provides an update of current literature on the ocular toxicity of metallic ENMs in vitro and in vivo, as well as the risks and benefits of therapeutic applications of metallic ENMs in ophthalmology.

## 1. Introduction

Demand for engineered nanomaterials (ENMs) has dramatically increased in recent years [1], with products manufactured for diverse applications including paints, electronics, clothing, cosmetics, and therapeutics, but the development of reliable safety assessments for ENMs has lagged behind their rapid commercialization [2]. These materials are engineered with particle sizes between 1 to 100 nm in at least one dimension, imparting functional advantages due to the unique physicochemical properties of nanoscale materials. The incorporation of ENMs into such a diverse array of products is driven by the unique and beneficial properties arising from their extremely small (<100 nm) size in at least one dimension. With their small size, the behavior of nanomaterials often diverges from that of their corresponding bulk material, thus making consistent and accurate predictions of ENM risk to human and environmental health challenging. Metallic ENMs are regularly incorporated into consumer products. While metallic ENMs may not be hazardous, their widespread use should not be taken as evidence that these substances are safe [3]. The increasingly ubiquitous presence of metallic ENM in the environment, water, soil, and air [4], increases the potential to cause toxicity to numerous organ systems [5]. There are multiple routes of entry for ENMs that depend greatly on the application and may be intentional or accidental. For example, nanomaterials may be injected intravenously for therapeutic, imaging, or diagnostic applications [6], absorbed dermally from cosmetics [7], ingested orally [8], exposed extra-ocularly or intra-ocularly [9,10], absorbed through inhalation [11], or produced as a result of undesired tribology—the science of wear, friction, and lubrication—consequences that lead to industrial and environmental contamination by ENMs [12]. Inhalation and dermal routes of exposure are especially prominent for gases, aerosols, and liquid particles containing ENMs [13]. Moreover, ENMs can be absorbed through the skin, accumulate in the lymph nodes, and from there spread to other organs [13,14,15].

The eye, particularly the cornea, experiences similar exposure to ENMs as skin and airways but may react to exposure in unique ways that differ from other tissue and organ types due to the immune privileged status of the cornea and the necessity of transparency for proper visual function. For example, a common response to ENMs exposed through the airway is inflammation [11,16]. However, it is unclear from the literature how ocular tissue responds to ENM exposure, as most literature focuses on the therapeutic possibilities of ENMs. There is a paucity of research into the risks that metallic ENM exposure poses for the eye. It is critical to assess ocular risk given that humans rely on high-acuity vision to navigate their environment. Vision impairment and blindness are a huge economic burden to individuals, caregivers, and society [17,18]. The eyes are particularly vulnerable to metallic ENMs applied in close proximity, such as zinc oxide (ZnO) and titanium dioxide (TiO_2_) that are commonly incorporated into commercial sunscreens and cosmetics [19,20]. ENMs are also a focus for ocular drug delivery, where nanodrug carriers are used to enhance drug permeability and control drug release within the eye [21,22,23,24,25]. The purpose of this article is to review (1) the characteristics and general toxicity of ENMs, (2) the relevant ocular anatomy pertaining to ENM exposure, and (3) the published literature regarding ocular toxicity.

## 2. Characteristics and General Toxicity of Metallic ENMs

Metallic ENMs are nanomaterials based on stable metals in the Periodic Table with at least one external dimension between 1–100 nm [26]. Metallic ENMs are normally divided into two categories: (i) metal, and (ii) metal oxides. Metallic ENMs can also be classified based on their shape, such as spherical, nanowires, nanorods, sheets and nanoplatelets [27]. Metallic ENMs have a natural tendency to aggregate and agglomerate and make larger structures than bulk materials, primarily due to their large surface energy [28]. Additionally, the presence of culture media and/or cells, which are highly ionic, can significantly alter the behavior of nanosized materials and induce aggregation and surface fouling due to protein adsorption [29,30]. Therefore, the stabilization and dispersion of metallic ENMs in a liquid medium is important for the study of ocular toxicity and delivery of metallic ENMs, as well as other in vivo and in vitro experiments. To address this stability concern, two main strategies have been utilized—electrostatic and steric stabilizations. In electrostatic stabilization, ENMs are stabilized by an electrical double layer formed by absorption of negatively charged ions [31]. This charged layer can repel individual ENMs from one another, preventing further agglomeration. The strength of this electrical double layer interfaced with ENMs is measured by a parameter called zeta potential [32]. Zeta potential can be tuned by pH, ionic strength, or temperature to obtain required stability. However, this method only works in polar liquids that can dissolve electrolytes, such as water or ethanol. The steric stabilization involves capping of the metallic ENMs with polymers, surfactants, or ligands, to the particle surface [33]. The long protruding chains of these organic molecules prevent individual particles from aggregation. Additionally, these chains push away any particles that approach each other. For a more in-depth discussion of the chemistry driving these unique interactions we refer readers to a review by Mu et al. [34].

Numerous in vivo studies have demonstrated that metallic ENMs can induce toxicity in a variety of organs including brain, heart, lung, liver, skin, kidney, and reproductive organs [35,36,37,38]. Toxicity may be related to the unique tendency of ENMs to accumulate at specific sites within organs, or at sites of initial exposure. We refer the reader to a review by Ema and coworkers [39] that examined the effects of silver ENMs (AgENMs) in laboratory animals and concluded that AgENM accumulation in the testis, sperm, ovary, embryo, placenta, and breast resulted in toxicity to these tissues. An in vivo study with zinc oxide (ZnO) ENMs of various sizes (20 and 100 nm) showed sub-chronic oral toxicity which lasted for up to 90 days in rats where toxicity was manifested as weight loss and alterations in liver enzymes [15,40]. Finally, increased expression of inflammatory cytokines such as TNF-α, IL-10, IL-12, IFN-γ, and/or NF-κB is common after dietary exposure to AgENMs (20 nm) and ZnO ENMs (20–100 nm) [40,41].

Metallic ENMs have received considerable attention because they have attractive antimicrobial properties through the generation of reactive oxygen species (ROS), metal-ion release, particle internalization into bacteria, and direct mechanical destruction of bacterial cell wall and/or membrane [42]. Unsurprisingly, these beneficial antimicrobial properties can also impact eukaryotic cells and account for the majority of the toxicological effects observed [35]. Consequently, the toxicity of metallic ENMs has been well-studied in numerous organs [5,39,42], and predominantly occurs via impaired cell viability, cellular uptake [43], inflammation [44,45], ROS related effects [44,46], DNA damage, cell cycle arrest, and cell death via apoptosis or necrosis [43,45]. These observed toxic effects were suggested to be non-specific interactions of ENMs with cells due to their chemical composition, surface coating and/or release of cations [47]. For example, AgENMs surface coated with polysaccharides induced greater DNA damage to mouse embryonic stem cells and fibroblasts than uncoated AgENMs, since the coated AgENMs agglomerated less thus enhancing penetration into cells [48] or via receptor mediated endocytosis. Various conformations of protein corona on the surface of ENMs also increase their access to membrane bound organelles for cellular uptake [49,50]. The features that make ENMs so effective as therapeutics are the same features that can lead to toxicity and other negative effects, adding layers of complexity to reliably determining the safety of metallic ENMs, both in vivo and in vitro.

## 3. The Eye Is a Formidable Barrier

**Figure 1:**
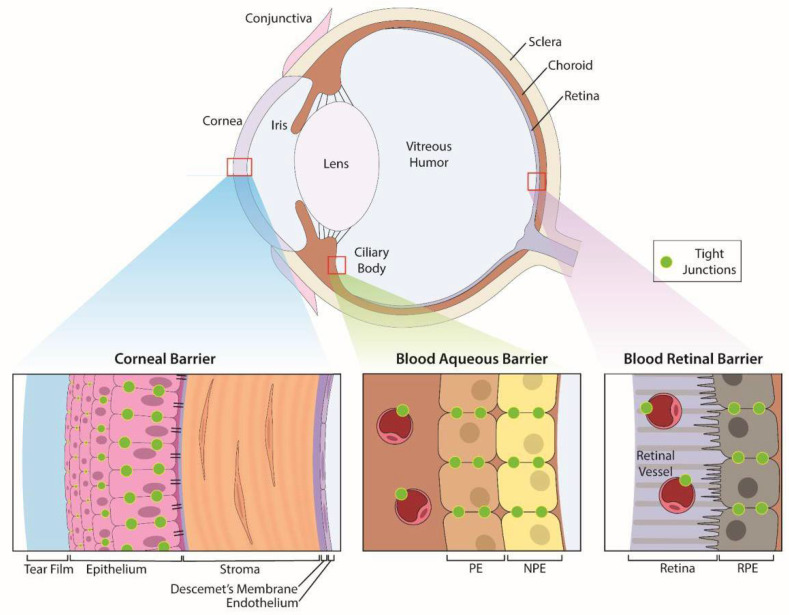
**Anatomic structures of the eye and its specific ocular barriers.** The eye has three layers that contain barriers: (1) the outer protective coat of the eye which consists of the cornea and sclera. (2) a middle, vascular layer consisting of the iris, ciliary body and choroid, and (3) the inner, neural coat comprised of the retina. The cornea provides protection of the inner structures of the eye with a transparent trilaminar sandwich comprised of a hydrophobic multilayered epithelium, which contains tight junctions between the apical cells, a hydrophilic stroma and a hydrophobic endothelium with its specialized basement membrane, Descemet’s membrane. Within these layers are the blood-aqueous barrier (BAB) and blood-retinal barrier (BRB) which limit penetration of infectious agents and toxins from the systemic circulation. The BAB consists of the non-pigmented epithelium of the ciliary body and the non-fenestrated endothelium (PE) of the iridal blood vessels while the BRB is comprised of tight junctions between cells of the retinal pigment epithelium as well as between retinal vascular endothelium.

Eyes are sensory organs specifically designed to provide vision. The eye is comprised of three layers—an outer, protective fibrous coat (cornea and sclera), a middle, vascular layer (iris, ciliary body and choroid), and inner, neural coat (retina) (Figure 1). The anterior segment of the eye contains structures in front of the vitreous humor (cornea, iris, ciliary body, and lens) and the posterior segment contains the vitreous humor, retina, choroid, and optic nerve.

### 3.1. Surface Barriers Prevent Exogenous Agents from Entering the Eye

The precorneal tear film covers the ocular surface and serves as the first functional barrier by diluting and slowing the penetration of any agent, including drugs or toxic agents, that contact the eye. Any exogenous material, including ophthalmic solutions, are diluted by tears, and typically removed during the first 30 s after instillation from the ocular surface via reflex tearing, blinking and drainage through the nasolacrimal duct [51]. For topically applied therapeutics, typically <5% of the applied dose makes it to the ocular surface [52]. This process of dilution by the tear film, and clearance via blinking and nasolacrimal drainage, presents challenges and a barrier to treating pathologies of the anterior segment of the eye. In addition to the corneal tear film, there are other obstacles within the eye that limit drug delivery, such as the blood aqueous barrier (BAB) [22]. The small size and novel properties of ENMs provide a promising platform for bypassing these barriers and targeting drugs for difficult to treat tissues and diseases, such as dry eye [53,54], age-related macular degeneration [55,56], glaucoma [57], and others [58,59]. However, the toxicity of these formulations remains under studied [60].

**Figure 2:**
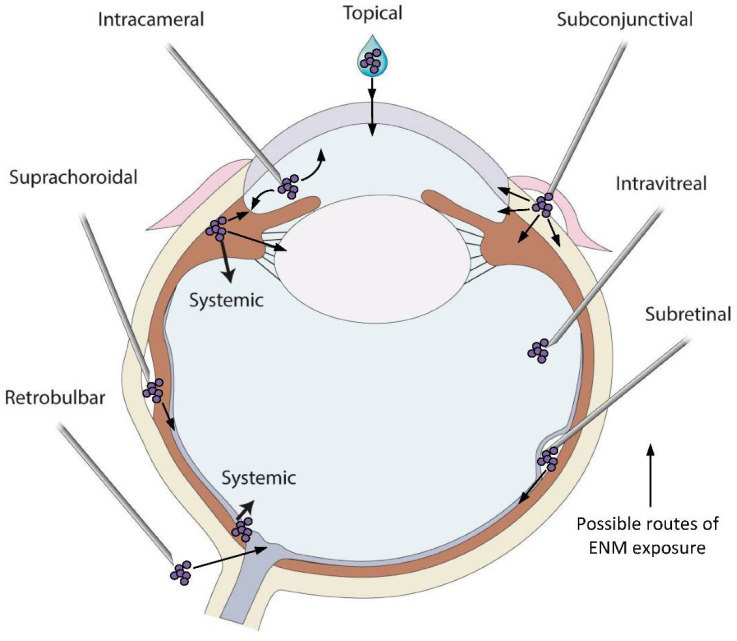
**Variety of drug application sites into the eye can be possible exposure routes of the metallic ENMs.** Topical application is the simplest method to deliver drugs and ENMs to the cornea and anterior chamber and the most common exposure route of metallic ENMs in environment. Systemic administration of drugs can be used to deliver drugs when the BAB/BRB is compromised or if the size of the drug is small. Intracameral administration requires injection of a substance directly into the anterior chamber to bypass corneal barriers. Subconjunctival injection, whereby a needle is inserted into the space between the conjunctiva and the sclera, is an alternative method to bypass corneal barriers. Intravitreal, subretinal and suprachoroidal injections are all used to deliver drugs to the posterior segment of the eye. Intravitreal injection is a commonly used delivery route to administer medications to treat a variety of retinal conditions. Subretinal injection is used to target more specific retinal cell types such as photoreceptors or retinal pigmented epithelial cells and is often used for gene delivery to those cells. Injections into the suprachoroidal space, a narrow space lying between the choroid and sclera extending from the limbus to the optic nerve, are used to effectively deliver pharmacologic agents to the retina and choroid directly. While intravitreally injected drugs spread diffusely across all parts of the eye, those injected via the suprachoroidal route rapidly distribute through the choroid and retina resulting in a higher local drug concentration. Retrobulbar injection is an injection in the retrobulbar space, the area located behind the globe of the eye, and a common way to target structures in the orbit. Through the administration routes listed here, all tissues of the eye have the potential to be exposed to metallic ENMs, with the biological response being highly dependent on the physicochemical properties of the ENMs in the specific in vivo environment. An ENM introduced to the vitreous behaves differently than an ENM introduced to the anterior chamber, or to the subretinal space. Care and consideration for the change in ENM aggregation and agglomeration in these vastly different environments must be taken.

The outermost fibrous coat of the eye, the cornea and sclera are in direct contact with the external environment and provide protection for the middle vascular and inner neural structures. The transparent cornea is a trilaminar sandwich comprised of a hydrophobic multilayered epithelium, a hydrophilic stroma and a hydrophobic single-layer endothelium with its specialized extracellular matrix, Descemet’s membrane (Figure 1). The outermost layer of the cornea is a continuously renewing epithelium that is routinely exposed to chemical, physical, and pathological agents [61]. This smooth and transparent epithelium limits permeation via the tear film and, secondly, via tight junctions between the apical epithelial cells [52]. Tight junctions are dynamic [62] and complex barriers that regulate permeability based on size and charge [63] through either a 4–8 Å pore pathway or a 3–10 nm leak pathway [64,65,66,67,68,69]. The sizes of these junctions are smaller than most metallic ENMs but the permeability of these junctions is sensitive to factors such as environmental pollution [67], diabetes [68,69,70], infection [71], and other factors [72,73,74]. Administration of drugs to the eye often relies on transcorneal penetration (Figure 2) and the ability of ENMs to penetrate these ocular barriers (Figure 1), which greatly depends on their size, composition, and charge. Topical administration is the simplest delivery route but only targets the anterior structures of the eye, with only a few drugs able to reach the vitreous humor, retina, and choroid at biologically relevant concentrations [75]. Despite these formidable barriers, some ENMs penetrate easily through the cornea and into the anterior chamber and consequently ENMs could be used as efficient carriers for drug delivery. For example, the high surface-area to volume ratio of nano-drug carriers may improve interaction with the outer mucin layer of the corneal epithelial surface thus promoting drug retention [76]. We refer readers to the strategies by which this occurs in a comprehensive review by Souza and co-authors [77] and a review by Janagam et al. [22] addressing nanoparticle design strategies for corneal penetration.

**Figure 3:**
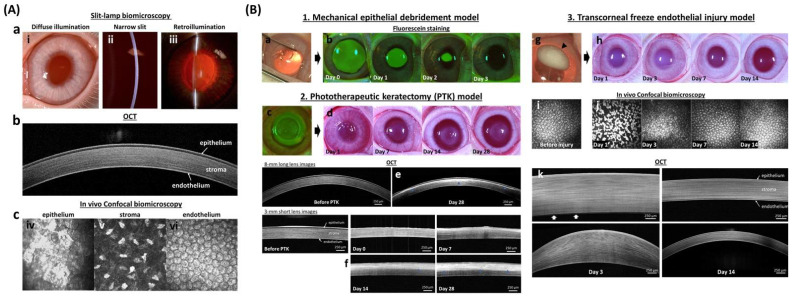
**In vivo corneal wound healing model in rabbits.** (**A**) In vivo imaging techniques of the normal cornea demonstrate the individual layers with which it is comprised. Digital slit-lamp biomicroscopy (**a**) is used to examine the anterior segment of the eye with diffuse illumination (**i**) used to examine the surface of the eyelid, cornea, and iris. A narrow slit-beam (**ii**) provides a cross-sectional image of the cornea and anterior chamber to determine the depth and character of corneal opacities as well as assess for intraocular inflammation termed and aqueous flare, respectively. Retroillumination (**iii**) demonstrates opacities of the cornea, anterior chamber or lens. Advanced ocular imaging is used to augment slit lamp biomicroscopy with optical coherence tomography (**b**) used for cross-sectional corneal imaging, while in vivo confocal biomicroscopy (**c**) shows individual cells of each corneal layer including the epithelium (**iv**), keratocytes in the stroma (**v**), and endothelium (**vi**). (**B**) Wound healing models for each corneal layer are well optimized to study both toxicity and drug efficacy. The mechanical debridement model (**1**) is used for corneal epithelial wound healing. The wound area, after removing corneal epithelium (**a**), can be visualized with sodium fluorescein stain which binds to the hydrophilic stroma (**b**). A phototherapeutic keratectomy (PTK; **c**) model (**2**) is used to evaluate the development of corneal scarring following stromal wounding. Digital images (**d**) show developed stromal opacity (haze) from day 14 to 28 after wounding. The depth of the corneal opacity in the corneal stroma can be visualized with hyperreflective area (blue arrowhead) using OCT (**e**,**f**). The transcorneal freeze injury model (**3**) is used to evaluate corneal endothelial repair. Digital images show ice ball (black arrowhead) formation right after the cryoinjury (**g**) and transient stromal edema (**h**) at day 1 and 3. Endothelial cell morphology can be examined in vivo using confocal biomicroscopy before (**i**) and after the injury (**j**) while OCT (**k**) is used to measure corneal thickness and thus assess the primary function of endothelial cells to maintain corneal deturgescence. White arrows (**k**) indicated bare Descemet’s membrane at day 3. The fluorescein images in section B 1. was reprinted with permission from [82]. 2021, Elsevier. All other images are unpublished data supplied by the authors.

In addition to serving as a barrier against foreign material, the cornea protects against mechanical trauma; thus, each layer must be able to heal with tight junctions reforming following wounding. In vivo corneal wound healing can be easily visualized and monitored using multimodal imaging techniques that specifically target each layer (Figure 3). The unique attributes of the cornea, particularly its transparency, offer advantages to study the interaction of metallic ENMs with tissues in vivo. Specifically, the ease of access to the cornea and its anatomical characteristics make a good experimental model to determine the impact of ENMs on wound healing, an under studied topic in nanotoxicology. The most common in vitro models for wound healing are 2D and include the scratch, stamp, thermal, electrical, and optical wounding of confluent cells, and observation of migration into the cleared areas [78]. These tests are convenient and useful screening tools that take advantage standard lab equipment and cultivation methods but are less applicable to in vivo 3D epithelial tissues. However, there are developments in 3D in vitro wound healing assays in the cornea [79], skin [78], and even high-throughput “wound on a chip” models [80,81] that may provide applicable and predictive results for future studies.

### 3.2. Blood Ocular Barriers Limit Access of Systemic Agents to the Eye

Within the eye, two blood ocular barriers exist to protect the eye from toxic agents within systemic circulation, a function that can be circumvented [83] (Figure 1). The BAB consists of the non-pigmented epithelium of the ciliary body and the non-fenestrated endothelium of the iridal blood vessels. Tight junctions between adjacent cells of the BAB provide a barrier that prevents most protein and cells of the plasma from entering the aqueous humor, the transparent fluid that fills the anterior chamber [84]. Similar to the BAB, the BRB is comprised of tight junctions between cells of the retinal pigment epithelium (RPE) as well as those of the retinal endothelial capillaries [76]. The BRB regulates ion, water, and protein transportation into and out of the retina and restricts the entry of large molecules, including many drugs.

Due to their small size, systemically administered metallic ENMs can circumvent these barriers and subsequently accumulate in various tissues and fluids in the eye [85]. While oral or intravenous administration is appropriate for some therapeutics to penetrate the BRB and deliver drug to the posterior structures of the eye, alternative delivery routes are necessary for those therapeutics that cannot readily cross these barriers. These alternative routes include intravitreal, subconjunctival, and subretinal injection, with intravitreal injection being the most widely used method (Figure 2). However, it is possible for some ENMs to circumvent the BRB. For example, intravenous administration of 20 nm gold ENMs (AuENMs) in mice allows them to pass through the BRB and, therefore, may improve the bioavailability of therapeutic agents for retinal disease [86]. Nanomaterial drug formulations that enable sustained drug release could reduce the frequency and increase the efficacy of intravitreal administration. For further information on the use of nanomaterials to treat retinal disease, we direct the reader to detailed reviews by Jo and colleagues [87] and Wu and colleagues [88].

Intravitreal injection is a common delivery route for therapeutics to the posterior segment of the eye. Consequentially, the bulk of existing research regarding toxicity and therapeutic applications of metallic ENMs has focused on this administration route as well as being limited in focus to gold, silver, iron oxide, and cerium nanoparticles (NPs) [10,89,90,91,92,93,94] due to their attractive therapeutic properties. However, neurological concerns of eye-to-brain transmission of silver (Ag) and titanium dioxide (TiO_2_) have been raised in the literature [95], highlighting a lack of research into the ability of a wide variety of metallic ENMs to cross these critical blood ocular barriers.

## 4. Toxicity and Utilization of Metallic ENMs in Ophthalmology

Herein, we discuss the peer-reviewed literature regarding the use of metallic ENMs in ophthalmology and their toxic effects to the eye.

### 4.1. Gold ENMs

AuENMs have been broadly studied as a therapeutic for the eye as well as for potential toxicity to ocular tissues [96]. Interestingly, toxicological evaluations observed in vitro often differ from those observed in vivo. Toxicities observed in the posterior segment of the eye do not necessarily predict what occurs in the anterior segment, given the anatomical differences between the two regions. Exposure to AuENMs produced a variety of adverse events that depended on the shape, sizes, delivery route, cell or tissue type, and functionalization of the AuENMs [86,97,98,99,100,101,102,103]. Below we summarize the literature on ocular toxicity of AuENMs.

#### 4.1.1. Anterior Ocular Toxicity

Few studies have examined the impact of AuENMs on the anterior segment of the eye, but the impact of particle size and nature of functionalization on toxicity remain central. One study using primary human corneal fibroblasts demonstrated the relationship between functionalization and size of AuENM on cellular toxicity. Corneal fibroblasts treated with polyethylenimine-conjugated AuENMs (~4 nm) exhibited a maximum decrease in cellular viability of 13% [103]. These AuENMs were also used in an in vivo rabbit model and were applied topically after corneal epithelial removal and examined 3 days after treatment. There was no effect observed on epithelial wound healing, but initial stromal cell death was higher than control eyes, with stromal cell counts returning to control levels after 7 days, indicating a delay in keratocyte repopulation [103]. While the Au core of these particles is quite small, and has been associated with cytotoxicity, the coating is a bulky 2 kDa polyethylenimine and is likely modulating toxic effects in the cornea [103]. Of note, Sharma et al. also followed AuENM concentration in the cornea for a month, with a peak concentration of 332 ppm at 12 h and persisting at 256 ppm at one month post treatment. These studies demonstrated an acute toxicity of AuENMs to the anterior segment of the eye, particularly to corneal stromal cells. The effects of long-term persistence of functionalized AuENMs within the cornea remain poorly understood.

However, not all AuENMs are toxic. In contrast to the above study, transfection of the *BMP7* gene using PEI2 coated AuENMs modulated corneal wound healing in a rabbit photorefractive keratectomy (PRK) model with minimal cytotoxicity and inflammation [104]. In another study, citrate-capped 15 nm AuENMs were utilized as a negative control in examining the cytotoxicity of various ENMs to human telomerase-immortalized corneal epithelial (hTCEpi) cells, with no cytotoxicity observed with calcein-AM or MTT assays [105]. The broad variety of ENM functionalization available to researchers makes predicting toxicity difficult.

#### 4.1.2. Posterior Ocular Toxicity

There has been a large focus on the use of AuENMs in the posterior segment of the eye, and in the retina specifically. Interestingly, several of the studies on the posterior segment of the eye demonstrate that trend of higher surface area correlates with higher toxicity. As an illustration, in two solutions with the same mass of small or large ENMs, the solution with small particles has more particles overall and, therefore, more surface area than the solution with large ENMs. Additionally, the greater the ratio of surface area to volume, the higher the chemical reactivity [106,107]. The increased toxicity with decreasing particle size is likely due in no small part to the intrinsic reactive properties of nanoscale materials. It is also important to remember that ENM behavior in culture media changes, as the presence of solutes can induce aggregation, agglomeration, and some solutes can physically and/or chemically interact with the ENM. Indeed, it is these interactions that likely impart either a toxic or beneficial effect. To highlight these phenomena, we present here several studies on the posterior section of the eye [105]. In one study, the toxicity of a variety of sizes (5–100 nm), shapes (spheres, cubes, and rods), and concentrations (0.01–5 mg/mL) of AuENMs were examined using ARPE-19 cells [98]. Further information on the ARPE-19 cell line can be found in [108]. Viability was defined as their ability to maintain growth and was evaluated using the MTT assay, with spheres under 30 nm and rods 10 × 90 nm adversely impacting viability even at low concentrations. In the aforementioned study, the authors found larger AuENMs (>50 nm) were poorly internalized, while smaller ENMs were readily taken up by the cells. Not only do smaller ENMs have greater access to the cell interior, but they are also more reactive once inside. Two additional AuENM shapes have also been investigated for retinal cell toxicity, flower (40 nm core) in ARPE-D407 cells [99] and nanodisks (160 nm wide, 20 nm thick) in human retinal microvascular endothelial cells (HRMECs) [109]. The flower shaped AuENMs showed concentration dependent toxicity, whereas the larger nanodisks showed low toxicity even at higher concentrations. Collectively, these in vitro results focused on the retina make a strong case for the importance of size and surface area of AuENMs on toxicity.

**Figure 4:**
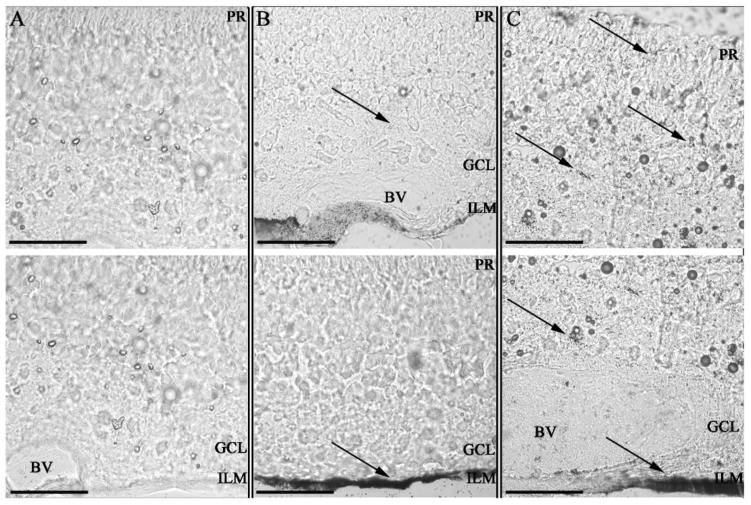
Impact of Hyaluronic acid coating on tissue invasion in porcine retinal explants. Retinal explants imaged with Confocal bright field microscopy at 63× magnification after silver staining with 100 nm scale bars; ILM = inner limiting membrane; GCL = ganglion cell layer; PR = photoreceptor layer and BV = blood vessel. Two sequential histological porcine retina per sample. Control non-treated retina (**A**) retinas 24 h after administration of GNPs (**B**) and HA-GNPs (**C**) at a concentration of 0.5 mM. Gold ENMs show a dotted black pattern inside the tissues with accumulation occurring at the administration point in the ILM and spreading to other tissues only for those ENMs functionalized with hyaluronic acid. This work by Apaolaza et al. highlights the impact ENM functionalization can have on biodistribution and subsequent opportunities for toxicity. Reprinted with permission from [97]. 2020, Elsevier.

The anti-angiogenic properties of AuENMs have made them prime candidates for evaluating ocular therapies for various retinopathies, resulting in several in vivo studies in the posterior segment. While not explicitly focused on toxicology, these studies demonstrate that the size and functionalization of AuENMs impact their toxicity to various ocular structures. Large ENMs (<100 nm) that were administered intravenously were unable to cross the BRB [83] and an in vivo study in rabbits demonstrated minimal retinal and optic nerve toxicity when large AuENMs (<220 nm) were injected intravitreally [92]. Similarly, large nanodisks (160 × 20 nm) were injected intravitreally into mice with oxygen-induced retinopathy and no inflammation, apoptosis, or changes in outer retinal function as measured with electroretinography, and were observed at five weeks post-injection [109]. Smaller ENMs (20 nm), however, were able to penetrate the BRB and dispersed throughout the retinal layers after a single intravenous injection [86]. It is likely that shape and functionalization also play a role in the ENM’s ability to cross these critical ocular barriers. The penetration and toxicity of small AuENMs (20 nm) was modulated through functionalization with hyaluronic acid (Figure 4), which demonstrated enhanced vitreous and retinal penetration in an ex vivo porcine eye model [97]. A different study showed that rabbits subretinally injected with AuENMs (12 nm) functionalized with goat-IgG showed mild retinal degeneration [100]. These results indicate that in vivo results may deviate from those in vitro, with size and particle functionalization exerting nuanced effects on toxicity to the posterior segment of the eye.

Aggregate these studies, and others (Table 1) (several of which have been well summarized in the review by Masse et al. [90]), suggest that the biocompatibility of AuENMs varies based on size, shape, and functionalization. Additionally, we observe that in vitro studies can inform, but may not directly translate to, in vivo ocular toxicity or biodistribution of AuENMs. Given that the diversity of AuENMs shape and size, as well as their variable chemical functionalization, exhibit differing impacts on cellular and tissue toxicity. Further dedicated testing of the safety profiles for AuENMs, particularly under diseased conditions, are required.

### 4.2. Silver ENMs

The AgENMs found in personal care products, as well as medical, textile, and antimicrobial products [110], can lead to direct contact with the ocular surface. Similar to AuENMs, AgENMs can also inhibit neovascularization and are gaining more attention and use in clinical care and diagnostics [101]. However, research into the cytotoxicity and adverse effects of AgENMs on ocular tissue is under studied, with investigations of a few AgENMs to various ocular cells having been evaluated in vitro and only to a limited extent ex vivo and in vivo (Table 2).

#### 4.2.1. Anterior Ocular Toxicity

The antimicrobial properties of Ag make AgENMs an enticing material for the control of microbial populations in individuals with long-term contact lens use. One study indicated low cytotoxicity of three different shapes of AgENMs capped with cathelicidin peptide for incorporation into collagen hydrogels to be used for corneal replacement or bandage contact lenses [111]. These data suggest that there is no acute cytotoxicity of AgENMs on corneal epithelial cells, but it would be beneficial to examine the corneal biodistribution of AgENMs, persistence in the tissue, and cytotoxicity to other corneal cell types.

**Figure 5:**
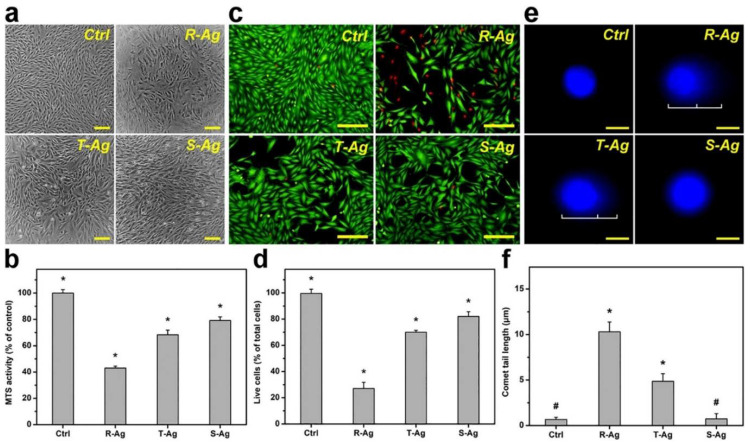
In vitro biocompatibility of three different shapes of AgNPs of similar size. Biocompatibility demonstrated in (**a**) Phase-contrast micrographs, (**b**) MTS activity, (**c**) fluorescence photomicrographs, and (**d**) live cells (Live/Dead assay), of Rabbit Corneal Keratocytes (RCK) cultures after a 2-day exposure to Rod shaped (R-Ag), Triangular shaped (T-Ag), and spherical shaped (S-Ag) nanoparticles at a concentration of 5 × 10^10^ particles/mL. The (**e**) fluorescence photomicrographs of the comet assay, and (**f**) comet tail lengths were exposed to the three different AgNPs for 24 h at the same concentration. The controls without AgNPs are present for each experimental condition. Scale bar in (**a**) is 50 μm, (**c**) 50 μm, and (**e**) 10 μm. * *p* < 0.05 vs all groups; # *p* < 0.05 vs R-Ag and T-Ag groups. Values are mean ± SD (*n* = 4). Reprinted with permission from [112]. Elsevier, 2021.

In contrast to corneal epithelial cells, corneal stromal cells do experience acute cytotoxicity when exposed to AgENMs. The toxicity of three shapes (rod, sphere, and star) AgENMs was investigated in vitro with corneal stromal cells, with equal concentrations of rod-shaped ENMs exhibiting the most toxicity and spherical-shaped ENMs the least (Figure 5). The difference in toxicity of the spherical versus rod ENM is fascinating, as it corroborates the results and conclusions observed by other researchers on AuENMs, despite the difference in cell type (rabbit corneal keratocytes versus ARPE-19 cells).

These shape-based toxicity consistencies could potentially serve as predictors of ENM toxicity in a variety of cell types. The same researchers took these three shapes of AgENMs into an in vivo rabbit model of *Staphylococcus aureus*-induced keratitis as an antiangiogenic and bacteriocidal treatment. They found that spherical AgENMs induce the highest bacterial killing while rod shaped AgENMs suppressed deleterious blood vessel development more than the other shapes, albeit with unfavorable side effects such as corneal thickening [112]. In aggregate, these in vitro and in vivo data suggests that AgENM effects on ocular tissues can be modulated by shape in vitro, but that the results in vivo are a bit more subtle. These results indicate that a more thorough examination of the relationship between ENM size, shape, and surface area on toxicity in specific cell types and tissues is necessary, especially as researchers translate in vitro results to in vivo therapeutics. Other in vivo ocular toxicity studies of AgENMs have been performed, though the topic remains an area of limited study, with one study demonstrating a single topical application of 100 mg volume of 10 nm colloidal AgENMs into the conjunctival sac exhibiting no toxicity using the acute eye irritation test in normal rabbits [113]. In an in vivo rabbit model of filtration surgery for glaucoma treatment, the authors demonstrated that AgENM treatment of the bleb site resulted in fewer α-smooth muscle actin positive myofibroblasts, suggesting reduced fibrosis [114]. This study suggests that AgENMs may have application for numerous ocular diseases where fibrosis is an issue, such as corneal scarring, and assessment of their safety in vivo is critical.

#### 4.2.2. Posterior Ocular Toxicity

Markedly less research has focused on the cytotoxicity of AgENMs in the posterior segment of the eye. However, several sizes of AgENMs have been investigated, one of which utilized an organotypic tissue culture model of the murine retina to demonstrate neuronal toxicity with oxidative stress, apoptosis vacuole formation, and pyknotic cells being observed when treated with AgENMs (20 and 80 nm) [102]. Consistent with this study, similarly sized (20–50 nm) AgENMs inhibited cell survival in bovine retinal endothelial cells [115] in a size-dependent manner [116], with the smaller ENMs being more toxic. Likewise, another study demonstrated that AgENMs (25 nm) induced apoptosis in ARPE-19 cells in a dose-dependent fashion [117]. In contrast, AgENMs used to deliver dye for live cell imaging and for routine ophthalmic surgeries in vivo induced little decrease in cell viability in the murine retina [118].

While there is a lack of consensus regarding the impact of size, shape, and functionalization of AgENMs on cytotoxicity, hints of their influence can be seen, specifically with regards to smaller ENMs being more toxic. Further studies are needed to elucidate these relationships for the safe and efficacious development of ophthalmic therapeutics using AgENMs.

### 4.3. Metal Oxide ENMs

Other metallic ENMs, including cerium dioxide (CeO_2_), titanium dioxide (TiO_2_), zinc oxide (ZnO), and magnetic ENMs (mENMs), have also been investigated in ophthalmology (Table 3). Below we summarize the available literature on the toxicity of these other metallic ENMs. Discussion on the influence of size, shape, and functionalization is necessarily limited, since these topics have not been fully explored within the more commonly used Au and Ag ENMs. It is the authors hope that by highlighting the importance of these physicochemical properties, future studies in all ENMs of ocular importance will address these factors.

#### 4.3.1. Titanium Dioxide ENMs

Despite the widespread incorporation of TiO_2_ ENMs in cosmetics and sunscreens [19,20] routinely applied near the eye, limited investigations of the safety profile of TiO_2_ ENMs have been performed in the eye. In the anterior segment of the eye, TiO_2_ ENMs (25 nm) were observed to reduce single cell migration using human corneal limbal epithelial cells [119]. Similarly, two sizes of TiO_2_ ENMs (100 and 25 nm) showed no toxicity to hTCEpi cells but did inhibit migration at higher concentrations [105]. When human lens epithelial cells were treated with light irradiation concurrent administration of TiO_2_ ENMs, significantly greater apoptosis was observed [120]. Furthermore, the TiO_2_ ENMs inhibited lens cell growth and induced excessive ROS generation that ultimately led to irreversible cell damage and death. In an in vivo study, topical application of TiO_2_ ENMs (~140 nm) demonstrated transient, reversible conjunctival redness using the acute ocular irritation test in healthy rabbits [121]. Another in vivo rabbit study showed that TiO_2_ ENMs (<45 nm) caused ocular surface damage and resulted in a reduction in conjunctival goblet cell area compared to control eyes [122]. These results indicate inconsistent cytotoxicity, both in vitro and in vivo in the anterior portion of the eye and demonstrate that cytotoxicity should not be the only metric by which the safety of a metallic ENMs to the eye should be measured. Given the continuous need to replenish the corneal epithelium, inhibition of cellular migration and increased ocular surface irritation after exposure to TiO_2_ ENMs warrants further investigation in disease conditions, such as dry eye.

In the posterior segment of the eye, exposing embryonic zebrafish to TiO_2_ ENMs (12 nm) in their water did not delay development or induce retinal neurotoxicity [123]. Experiments on a variety of in vitro retinal cell types and intravitreal injection of TiO_2_ ENM in an in vivo mouse model showed a lack of toxicity [124]. This lack of toxicity is interesting, as TiO_2_ has been demonstrated to be readily taken up by ARPE-19 cells, as measured by flow cytometry and dark field microscopy [125]. However, a recent study demonstrated that while TiO_2_ ENM may not directly reduce cellular viability; they do induce degradation of claudin proteins, resulting in increased retinal endothelial cell migration in vitro. A single intravitreal injection of TiO_2_ ENMs has been shown to disrupt the BRB and impair normal retinal electrophysiology in the mouse [126]. These results are an interesting contrast to the inhibition of migration of corneal epithelial cells [105], further highlighting the effect of TiO_2_ ENMs on cell behavior while not exhibiting direct cytotoxicity. In aggregate, these results indicate that an ENM need not be directly cytotoxic to disrupt normal eye function, and warrant further investigation given the abundance of TiO_2_ ENMs routinely applied near the eye via cosmetics and sunscreens.

#### 4.3.2. Zinc Oxide ENM

In comparison to the previously discussed metallic ENMs, ZnO ENMs tend to be directly toxic to various tissues and cells of the eye [127]. In the anterior segment of the eye, ZnO ENMs (40–100 nm) inhibited cell viability and wound closure in vitro in a monolayer of immortalized human corneal-limbal epithelial cells from IK Gipson, Schepens Eye Research Institute (Boston, MA, USA) [119], and ZnO ENMs decreased cell viability in a concentration-dependent manner in Statens Seruminstitut Rabbit Corneal [128] (SIRC) cells [129] and in human tenon fibroblasts [130,131]. Similarly, we have shown that ZnO ENMs decrease cell viability and migration in vitro in hTCEpi cells, and that the presence of ZnO ENMs significantly delays wound healing (by 31–81 h) following epithelial debridement in an in vivo rabbit model [105]. These results indicate that ZnO ENMs are quite toxic to the anterior segment of the eye, particularly the cornea, and should be used with caution, despite their attractive antimicrobial properties. For example, ZnO ENMs incorporated in an antimicrobial media for daily use soft contact lenses [132] may negatively impact the corneal epithelium particularly following injury.

In the posterior segment of the eye, an in vitro study demonstrated that ZnO (5 nm) reduced cellular viability of both HCECs and ARPE-19 cells [133], and a murine photoreceptor-derived cell line (661 W) [134]. Other Zn based ENMs including zinc sulfide (ZnS, 50–200 nm), showed dose-dependent cytotoxic effects on primary mouse retinal pigment epithelial cells [135]. A 90-day in vivo study in rats demonstrated marked atrophy of the retina following oral gavage of 500 mg/kg ZnO ENMs (20–100 nm) [15,40]. These results suggest that not only are ZnO ENMs toxic to a variety of retinal cell types, they are able to penetrate the BRB and impact retinal health in vivo. Soluble ions, such as Zn^2+^, are likely to be the primary contributor to cytotoxicity of both ZnO ENMs and ZnS ENMs. A recent study demonstrated particle dissolution and release of toxic Zn^2+^ from ZnO in the cell culture medium with subsequent accumulation in the lysosomal compartment of mouse macrophage and human bronchial epithelial cell lines that triggered ROS generation and oxidative stress [136]. Consistent with these observations, ZnO ENMs (10–35 nm) triggered marked mitochondrial-induced cell apoptosis by promoting cytochrome c release, decreasing intracellular ATP and reducing the total antioxidant enzyme activities in murine photoreceptors in comparison to untreated controls [127].

#### 4.3.3. Cerium ENMs

Cerium containing ENMs (CeENMs) have been evaluated in vitro and in vivo for a diverse array of ocular applications, with CeO_2_ being the most well studied. As free radical scavengers, CeO_2_ ENMs show promise as a treatment for oxidative stress [137]. A variety of in vivo studies have examined the therapeutic efficacy of CeENMs, from ROS-protective effects in an age-related macular degeneration (AMD) mouse model [138], neuroprotective properties in a diabetic rat model [139], to a novel therapy for diabetic cataracts [140,141] where oxidative stress is a major issue [142], and lastly as a treatment to reduce corneal inflammation and opacification [143]. Only a few studies involving CeENM have directly addressed ocular toxicity, with none observed at low concentrations and higher concentrations of CeO_2_ (100 μg/mL) demonstrating putative genotoxicity in vitro in human lens epithelial cells [144,145]. We have examined the cytotoxicity of CeO_2_ (10 and 30 nm) in hTCEpi cells and did not observe a decrease in cell viability, but did observe reduced epithelial cell migration at higher doses [105]. In aggregate, these results demonstrate that while CeENMs seem to be non-toxic in vitro, they are being tested for a variety of ocular therapeutics applications, and there is a noticeable lack of in vivo safety testing. Therefore, detailed toxicity studies and long-term evaluations of the Ce ENMs are warranted.

#### 4.3.4. Other Metallic ENMs

We have previously screened a wide variety of metal oxide ENMs for in vitro toxicity using hTCEpi cells, include Al_2_O_3_, Fe_2_O_3_, CuO, V_2_O_5_, MgO and WO_3_ [105]. Of the materials tested, Fe_2_O_3_, CuO, and WO_3_ induced cytotoxicity at higher doses, with V_2_O_5_ significantly reducing cellular viability starting at a dose of 5 µg/mL. All of the materials, other than WO_3_, inhibited cell migration. This in vitro screening process allowed us to select promising candidates for in vivo testing, where V_2_O_5_ did not delay epithelial wound healing in a rabbit model [102]. Other authors have demonstrated the toxicity of CuO (50 nm), reporting the material impedes wound healing and cellular migration in both HCLE and HCF cells [119]. As the opportunities for human contact with other metallic oxide ENMs increases, either through commercial products or environmental contaminants, it will become necessary to scrutinize the impact of these materials on ocular health more closely.

#### 4.3.5. Magnetic ENMs

The magnetic properties of iron based magnetic ENMs (MENMs) are increasingly attractive for targeted drug and cell delivery to specific structures within the eye. The consequences of magnetic field exposure on cells are not well defined. In one study, MENMs were used to deliver mesenchymal stem cells to the trabecular meshwork as a treatment for glaucoma, with no adverse impact on cell viability and multipotency [146]. Additionally, there are several examples of MENMs being used to successfully target gene therapies using transfection to treat retinal vascular endothelial cells without ROS formation or cellular necrosis [147] and also deliver gene products without a viral vector due to the ability to encapsulate and functionalize MENMs [148]. Regardless of the injection route (into the anterior chamber or vitreous), MENMs (50 nm) were well tolerated by both corneal endothelial cells as well as retinal tissues [148]. MENMs can be targeted to certain tissues, such as the RPE of *Xenopus* and zebrafish embryos [149]; however, functionalization of the ENMs can alter the localization fate [150]. This is important as many therapeutic MENMs are functionalized with a drug of interest, and even if the functionalized MENMs demonstrate good biocompatibility [151] it is critical that the therapeutic arrives at the correct destination to reduce unwanted interactions. In a study utilizing superparamagnetic iron oxide nanoparticles (SPIONs), no inhibition of rabbit endothelial cell function in vitro was observed [152]. Additionally, in a separate study, these particles were used to guide bovine corneal endothelial cells to an injured area via an external magnetic force without impacting cell viability or disturbing the cytoskeleton [153].

In summary, these results demonstrate a putative role of MENMs as an ocular drug delivery platform; however, it is unclear how long these materials persist in the targeted tissue and the potential for degradation of any functionalized coatings could leave cells vulnerable to damage by the core of the ENM. Further studies are needed to fully characterize the toxicity of MENMs, especially under the disease states that are being treated.

## 5. Conclusions

We have reviewed the available peer-reviewed literature regarding ocular nanotoxicology and a handful of ophthalmic applications of metallic ENMs. Despite their promising utility for the treatment of a myriad of ocular diseases, numerous metallic ENMs showed variable and often unpredictable toxicity to cells and ocular tissues. Despite the push for metallic ENM therapeutics, there is a clear knowledge gap in the use, toxicity, and mechanistic evaluation of the biological effects (if any) of metallic ENMs in the eye. Therefore, comprehensive studies of the ocular nanotoxicology of metallic ENMs are clearly needed with the goal of identifying the underlying physicochemical properties of metallic ENMs that may be driving toxicity. Furthermore, specific to ocular surface applications, studies that focus on the effects of ENMs on corneal wound healing, barrier integrity, and tear film stability are critical, since the ocular surface is the front-line defence against environmental contaminants as well as a primary route for ocular drug delivery. The rise of ENMs in clinical ocular applications, such as imaging and treatments for diabetic retinopathy, also highlight the need for a better understanding of the biological effects of these ENMs on the eye. Deeper insights into the mechanism of toxicity in the eye will improve formulation of ENMs for therapeutic use, improving the safety and efficacy of metallic ENM-based therapies and result in better outcomes for our patients.

## Figures and Tables

**Table 1:** Published studies utilizing gold nanoparticles (AuENMs) in ophthalmology.

Metallic ENMs	Size (nm)	Synthesis/Stabilization	Characterization Methods	Concentrations	Cell Types/Animals	Treatment Times/Details	Experimental Design	Toxicology	Reference
**In vitro**									
IgG-absorbed AuENMs	12	Citrate reduction of HAuCl_4_	Spectrophotometer (maximum absorption at 520 nm), TEM	10 and 100 μM, 1 mM	Human RPE cells (ARPE-19)	24, 48, 72 and 96 h	Proliferation Curve (cell count with hemocytometer)	No significant differences in proliferation at all concentrations	Hayashi et al., 2009
AuENMs	20 and 100	Commercially purchased	Not specified	1, 10 and 100 μM/L	HRMECs and human retinoblastoma cells	48 h	MTT, ICC, Western blotting (ZO-1, glut-1, neurofilament)	No effect on cell viability or change in expression of representative biological molecules (ZO-1, glut-1, neurofilament)	Kim et al., 2009
Au-Nanoflower	40 (gold core) with 10 nm protrusions	Synthesized with _L_-ascorbic acid and HAuCl_4_	Spectrophotometer, TEM	0.47–5.64 × 10^−13^ M	Human RPE cell line	24 h	MTT	Significantly lower cell viability at ≥0.47 × 10^−^^13^ M	Boca et al., 2011
AuENMs	20	Commercially purchased	Not specified	0.1–10 μM	HRMECs	48 h	MTT, Wound migration, tube formation assay, Western blot (VEGFR-2, ERK1/2)	No toxicity observed with all assays	Kim et al., 2011
PEI2-AuENMs	Not specified	Synthesized by conjugation of thiol modified 2-kDa PEI to AuNPs	Not specified	150 mM (1.9 to 6.5 μL)	Primary human corneal fibroblasts	1 h treatment/24 h without NPs	Trypan blue exclusion assay, transfection AuNP-plasmid	Significant transgene delivery without altering the viability or phenotype of cells	Kim et al., 2011
AuENMs	20	Commercially purchased	Not specified	0.1, 1 and 10 μM	Human RPE cells	24 h	Apoptosis (cytotoxicity)	No cytotoxicity against RPE cells	Roh et al., 2016
Au-Nanodisks	160 in diameters; 20 in thickness	Top-down synthesis	SEM, Seta potential analysis, UV (830 nm) -vis measurement	1 and 3 pM/1–10^4^ particles per cell	HRMECs	12–48 h	WST-1, wound migration assay	No cellular toxicity; suppressed VEGF- induced migration of endothelial cells	Song et al., 2017
AuENMs	50	Synthesized by employing HAuCl_4_-gold halides	TEM, spectrophotometer	50–600 μg/mL	Melanoma cells (extracted from malignant choroidal melanoma patient)	24, 72 and 168 h	MTT, imaging and apoptosis detection after irradiation (30 Gy radiation)	Induce cytotoxicity a ≥200 μg/mL; AuNPs with irradiation induced melanoma cell apoptosis	Kanavi et al., 2018
Au-Nanorods	11 × 43	Commercially purchased	FESEM	Not specified	Y79 retinoblastoma cells and fetal retinal cells	1 h	MTS, Calcein-AM, propidium iodide fluorescence microscopy after scanned with femtosecond laser pulses (35 fs laser pulses at a central wavelength of 800 nm)	Au-nanorods induced cell ablation	Katchinskiy et al., 2018
Antisense hairpin DNA- functionalized AuENMs	37 ± 4	Synthesized	DLS	0–5 nM	Retinal microvascular endothelial cells	1–24 h	Live-dead assay, TEM	Detect and monitor VCAM-1 mRNA activity by TNF-α without acute toxicity	Uddin et al., 2018
Au-nanospheres with HA	20 nm gold core	Citrate reduction of HAuCl_4_.	UV-vis spectroscopy, PCS, LDV, TEM	25 µM and 50 µM	Adult retinal pigment epithelial cell line ARPE-19	2, 4, 6 and 24 h	Cellular uptake and distribution MTT measuring activity against AGE cytotoxicity	HA modified NPs do not inhibit AGE induced cytotoxicity compared to bare AuNPs	Apaolaza et al., 2020
**In vivo**									
AuENMs	Not specified	Adding sodium borohydride to HAuCl_4_ under vigorous stirring	Not specified	67 and 670 μM/0.1 mL	Dutch-belted rabbits	IVT; once; 1 week and 1 month	Histopathology (retinotoxicity)	No signs of retinal or optic nerve toxicity	Bakri et al., 2008
IgG-absorbed AuENMs	12	Citrate reduction of HAuCl_4_	Spectrophotometer (maximum absorption at 520 nm), TEM	10 and 100 μM, 1 mM	Rabbits	Subretinal inj.; once; 1 and 3 months	Fundus photo, IHC, TEM	Injected AuNPs were observed in the outer segments of photoreceptors at 1 month after the injection and were accumulated in the lysosomes in the cytoplasm of the RPE at 1 and 3 months after injection. Mild retinal degeneration and pigmentation with no cytotoxicity	Hayashi et al., 2009
AuENMs	20 and 100	Commercially purchased	Not specified	1 g/kg	C57BL/6 mice	IV (diluted in PBS); once; euthanize at 1 and 7 days after the injection	TEM, TUNEL, H&E	20 nm NPs passed through the BRB and were distributed in all retinal layers	Kim et al., 2009
AuENMs	20	Commercially purchased	Not specified	1 μM in 1 μL PBS	C57BL/6 mice	IVT; once; on P14; 3 days	Oxygen-inducedretinopathy; fluorescein angiography, TUNEL, H&E	Inhibit retinal neovascularization	Kim et al., 2011
PEI2-AuENMs	Not specified	Synthesized by conjugation of thiol modified 2-kDa PEI to AuNPs	Not specified	150 mM/100 μL	New Zealand White rabbits	Topical; 5 min at the central 7 mm cornea after epithelial debridement; 12 and 72 h or 7 days	Clinical exam, TUNEL, silver staining (distribution), instrumental neutron activation analysis (quantify the amount of AuNP uptake)	The PEI2-AuNPs were detected in the keratocytes and the extracellular matrix up to 7 days after topical application with no inflammation or redness and only moderate cell death and immune reactions	Sharma et al., 2011
AuENMs	30	Sodium citrate with HAuCl_4_ solution	Spectrophotometer (520 nm), TEM, XRD	40 mg/mL	Wister rats	Topical; q6 h; 24 h	Endotoxin (LPS) induced uveitis model; ELISA (TNF-α level), western blot (TLR4, NF-κB)	Anti-inflammatory effects (down- regulation of the TLR4-NF-κB pathway)	Pereira et al., 2012
TMAT-AuENMs	1.3 ± 0.4	Cation ligand (triphenylphosphine) stabilization	Proton nuclear magnetic resonance, UV-vis, TEM, small-angle X-ray scattering	0.08–50 mg/L	Zebrafish	0 to 120 hpf	Developmental toxicity, in vivo cell death (IHC, WISH, TUNEL, PCR), behaviour testing	Behavioural and neuronal damage in the developing zebrafish	Kim et al., 2013
PEI2_AuENMs	Not specified	Synthesized by conjugation of thiol modified 2-kDa PEI to AuNPs	Not specified	150 mM (with 10 μg of plasmid DNA)	New Zealand White rabbits	Topical; 5 min; 4 weeks	Photorefractive keratectomy (PRK); clinical exam, immunofluorescence staining (α-SMA), TUNEL,	PEI2-AuNPs showed substantial BMP7 gene delivery into keratocytes. Localized BMP7 gene therapy showed a significant corneal haze decrease and inhibits fibrosis without immunogenic effects and calcification	Tandon et al., 2013
AuENMs	20	Commercially purchased	Not specified	5 μL/drop	Balb/c mice	Topical; q6 h; 7 days	Alkali burn model; corneal neovasculazation analysis, Western blot (VEGFR2, ERK1/2)	Significantly reduced inflammatory corneal neovascularization by inhibiting the ERK pathway	Cho et al., 2015
AuENMs	20	Commercially purchased	Not specified	10 μM/1 μL	C57BL/6 mice	IVT; once; 2 weeks	CNV model; choroidal flat-mounts, IF (isolectin B4)	Inhibited CNV	Roh et al., 2016
AuENMs	30	Sodium citrate with HAuCl_4_ solution	UV-vis spectroscopy, XRD diffractometry, TEM	40 mg/mL	Wister rats	Topical; q6 h; for 24 h	Endotoxin (LPS) induced uveitis model l; ELISA, western blot for VEGFR2	No decrease in VEGF and VEGFR2 concentrations in the rat retina	Pereira et al., 2017
Au-Nanodisks	160 in diameters; 20 in thickness	Top-down synthesis (re)	SEM, Seta potential analysis, UV (830 nm) -vis measurement	1 and 3 pM	C57BL/6 J mice	IVT; once (P14); 3 days (P17)	Oxygen-induced retinopathy; VEGF measurement (ELISA), isolectin-B4 (retinal neovascularization), toxicity evaluation (Histology, TUNEL, ERG)	Attenuate neovascularization of oxygen- induced retinopathy without histologic or electrophysiologic toxicity	Song et al., 2017
AuENMs	50–100 nm	Citrate reduction of HAuCl_4_	TEM, zetasizer	0.025 mM loaded into contact lens	New Zealand white rabbits	GNP-modified contact lenses in both eyes, timolol-soaked lens in left, control in right for 4 days	Analyzing release of timolol in tear film, histopathology after 4 days (hematoxylin stain)	Normal nonkeratinizing epithelium observed	Maulvi et al., 2019
**Ex vivo, etc.**									
Au-Nanorods	10–15 in diameter/40–60 in lengths	Synthesized in a seed mediated approach	Spectrophotometer, TEM	10 nM colloids (<10% *w*/*v*)	Ex vivo porcine anterior lens capsule	Sandwich laser-welding	Photothermal effects of laser activated ENMs	Fusion of lens capsules with thermal damage	Ratto et al., 2009
Au-Nanocages	5 × 65	Synthesized by microwave assisted polyol methods	SEM, TEM, XRD, EDS	17–100%	Ex vivo porcine eye	High-contrast imaging conducted using tubing filled with solutions of different concentrations of Au-nanocages	Biological photoacoustic imaging and ultrasound imaging	Potential utility for diagnostic imaging of ocular disease	Raveendran et al., 2018
Au-nanospheres with HA	20 nm gold core	Citrate reduction of HAuCl_4_	UV-vis spectroscopy, PCS, LDV, TEM	0.5 mM	Ex vivo porcine eye	Vitreous separated and injected with 100 µL NPs for 24 h Applied to retinal explants for 24 h	Diffusion and localization of NPs observed with bright field camera (vitreous) or microscopy (retina, 12 µm cryosections) and TEM for retinal explants	Vitreous: aggregation 4 h post administration, no diffusion outside injection site Retina: distributed from ganglion cell layer to photoreceptors	Apaolaza et al., 2020

CNV, choroidal neovascularization; DLS, dynamic light scattering; EDS, energy-dispersive X-ray spectroscopy; ERK, extracellular signal-regulated kinase; FESEM, field emission scanning electron microscopy; HAuCl_4_, tetrachloroauric acid; HRMECs, human retina microvascular endothelial cells; Hpf, hour postfertilization; H&E, Hematoxylin and eosin; ICC, immunocytochemistry; IHC, immunohistochemistry; IV, intravenous injection; IVT, intravitreal injection; LPA, lipopolysaccharide; MTS, 3-(4,5-dimethylthiazol-2-yl)-5-(3-carboxymethoxyphenyl)-2-(4-sulfophenyl)-2H-tetrazolium, inner salt; MTT, 3-(4,5-dimethylthiazol-2-yl)-2,5-diphenyltetrazolium bromide; PBS, phosphate buffered saline; PCR, polymerase chain reaction; PEI, polyethyleneimine; RPE, retinal pigment epithelium; SAXS, small-angle X-ray scattering; SEM, scanning electron microscopy; TEM, transmission electron microscopy; TLR4, Toll-like receptor 4; TMAT, trimethylammoniumethanethiol; TUNEL, terminal deoxynucleotidyl transferase dUTP nick end labeling; VCAM-1, vascular cell adhesion molecule 1; VEGF, vascular endothelial growth factor; VEGFR-2, vascular endothelial growth factor receptor 2; WISH, whole-mount in situ hybridization; WST-1, water-soluble tetrazolium salt; XRD, X-ray diffraction; ZO-1, zonula occludens-1.

**Table 2:** Published studies using silver nanoparticles (AgENMs) in ophthalmology.

Metallic ENMs	Size (nm)	Synthesis/Stabilization	Characterization Methods	Concentrations	Cell Types/Animals	Treatment Times/Details	Experimental Design	Results	References
**In vitro**									
AgENMs	80	Not specified	EM, optical microscopy	40 mg/15μL	Retinal progenitor cells	NPs were propelled under 75–250 psi of pressure	Live/Dead Cell Viability/Cytotoxicity Kit	AgNPs were delivered rapidly and efficiently with minimal cell damage	Roizenblatt et al., 2006
AgENMs	20, 40 and 60	Commercially purchased	Not specified	2–10 μM (7 × 10^11^, 9 × 10^10^ and 2.6 × 10^10^ particles/mL suspensions)	Murine RAW264.7 cell line Transformed human corneal epithelial cells	1, 2 and 3 weeks	ToxiLight^®^ bioluminescence assay (toxicity), Bacterial viability, ELISA (IL-1β, IL-4, IL-6, IL-8)	Minimal microcidal and cytotoxic effects	Santoro et al., 2007
AgENMs	40–50	Synthesized using wet *B. licheniformis* biomass and 1 mM AgNO_3_ solution	DLS, spectrophotometer	100–500 nM	Bovine retinal endothelial cells	24 h	MTT, cell migration assay, Western blots, caspase-3-enzyme activity, DNA ladder analysis	AgNPs inhibit cell survival via PI3K.Akt dependent pathway	Kalishwaralal et al.., 2009
AgENMs	20–30	Commercially purchased	Not specified	0.0156 to 8 µg/mL	216 fungi strains (*Fusarium* spp., *Aspergillus* spp., and *Al. alternate*)	48 h at 35 °C	Antifungal susceptibility test	AgNPs exhibits potent in vitro activity against ocular pathogenic filamentous fungi	Xu et al., 2013
AgENMs (green and blue)	10–100	Synthesized using a modification of the photochemical preparation (Green AgNPs) or LED- mediated re-shaping methods (Blue AgNPs)	TEM, DLS	500 µM	Human corneal epithelial cells	12 h, 1, 3 and 5 days	Cell proliferation assay	No cytotoxicity observed	Alarcon et al., 2016
AgENMs nanorods	96 × 12 nm	Detailed synthesis for all shapes in publication	TEM, ICP, XRD	10 ppm and 5 × 10^10^ particles/mL	Rabbit Corneal Keratocytes	48 h	Morphology, MTS assay, Comet assay, DCFH-DA assay	Rod—lowest biocompatibility Sphere—highest biocompatibility	Nguyen et al., 2020
AgENMs (green and blue)	10–100	Synthesized using a modification of the photochemical preparation (Green AgNPs) or LED- mediated re-shaping methods (Blue AgNPs)	TEM, DLS	500 µM	Cornea-shaped collagen hydrogels (500 μm thickness) Incubation with *Pseudomonas aeruginosa*	Coating with Green or Blue AgNPs (12, 24, 72 h) 24 h	Mechanical testing (tensile strength, elongation), Light absorption, transparency, silver releasing rates (spectrometry) Measure survival colonies cultured after 24 h incubation	Blue AgNPs more transparent than normal yellowed colored AgNP in the hydrogel Survival colonies were reduced after exposure to Green-1 and Blue AgNPs	Alarcon et al., 2016
AgENMs nanorods nanotriangles nanospheres		Detailed synthesis for all shapes in publication	TEM, ICP, XRD	10 ppm	New Zealand white rabbits	72 h	Anti-corneal neovascularization with slit-lamp microscopy (maximum vessel length)	Rod—highest antiangiogenic activity Sphere—lowest antiangiogenic activity	Nguyen et al., 2020
				5 × 10^10^ particles/mL		72 h	Bacterial Keratitis clearing	Spherical AgNP induced complete clearing by day 3 postoperatively	

DLS, dynamic light scattering; ELISA, enzyme-linked immunosorbent assay; EM, electron microscopy; IHC, immunohistochemistry; IOP, intraocular pressure; MTT, 3-(4,5-dimethylthiazol-2-yl)-2,5-diphenyltetrazolium bromide; TEM, transmission electron microscopy; TUNEL, terminal deoxynucleotidyl transferase dUTP nick end labelling.

**Table 3:** Published studies utilizing other metallic ENMs in ophthalmology.

Metallic ENMs	Size (nm)	Synthesis/Stabilization	Characterization Methods	Concentrations	Cell Types/Animals	Treatment Times/Details	Experimental Designs	Results	References
**In vitro**									
CeO_2_ ENMs	6.3	Synthesized by adding H_2_O_2_ to cerium (III) acetate hydrate solution with the mixture being continuously stirred	TEM, High-resolution spectrophotometer	5 and 10 μg/mL	HLE cells (ATCC-LGC CRL- 11421)	24 h	Alkaline COMET assay (DNA damage)	No genotoxicity or DNA damage	Pierscionek et al., 2010
CeO_2_ ENMs	6	Synthesized by adding H_2_O_2_ to cerium (III) acetate hydrate solution	TEM, High-resolution spectrophotometer	10, 20, and 100 μg/mL	HLE cells	72 h	Alkaline COMET assay, Live cell imaging for cell growth	Potential genotoxicity at higher exposures; no impact on cell growth	Pierscionek et al., 2012
CeO_2_ ENMs	20 nm	Ce(NO_3_)_3_ added to buffer with Sodium acetate and ethylic acid and stirred before dilution, heating, and five cycles of centrifugation and resuspension	TEM, XPS	0-100 mg/mL	HCECs	24 h	MTT, migration, ROS (DCFDA assay), NO (Griess reagent)	CeNPs inhibited migration but exhibited no toxicity. Reduced ROS and NO production.	Zheng et al., 2019
	10 and 100 nm	Purchased							
Silica-CeCl_3_ ENMs	130	Stirred the mixture solution of micro-porous silica power material and CeCl_3_ powder by magnetic stirring	SEM, DLS	6 and 12 mg/mL	HLE cells	24 h	Intracellular ROS and GSH assay	Inhibited formation of advanced glycation end-products and reduced oxidative stress	Yang et al., 2014
TiO_2_ ENMs	60	Commercially purchased	TEM	2.5–10 µg/mL	HLE cells (HLE B-3)	24–72 h	MTT assay, measurement of ROS and intracellular Ca^2+^ level with UVB irradiation	Inhibit cell proliferation, generate excessive ROS and elevate the intracellular Ca^2+^ level; potential for the application of PCO treatment under UVB irradiation	Wu et al., 2014
TiO_2_ ENMs	36–97 nm	Commercially purchased	BET test, TEM, DLS, XRD (contracted outside lab)	0.1–30 µg/mL	ARPE-19 cells	24 h	Calcein-AM and propidium iodide, flow cytometry, and fixed cells stained with DAPI, HO3342, YoPro1, SYTOX green, and SYTOX orange	NPs localized to ER and surrounded nucleus and concentration dependent aggregates within cytoplasm. ~2% decrease in cell viability at highest dose. TiO_2_ NPs showed dose dependent changes in FSC and SSC intensity in flow cytometry.	Zucker et al., 2010
TiO_2_ ENMs	42 nm	Commercially purchased	TEM, DLS, zeta-potential	10–1000 ng/mL	HREC, ARPE-19	24 h	MTT	HREC cytotoxicity observed in dose dependent fashion, ARPE-19 not effected	Chan et al., 2021
TiO_2_ ENMs CuO ENMs ZnO ENMs	25 <50 40–100	Commercially purchased	Nitrogen adsorption/Bruanuer–Emmet–Teller (BET) method (characterize specific surface area), X-ray diffraction, DLS	≤108 μg/mL	HCLE cell line, HCFs	18 h	MTT and Alamar Blue assay (cell viability), CyQUANT^®^ assay (Cell proliferation), Circular wound healing bioassay, Single cell migration assay, Cellular uptake	CuO impeded wound healing of HCLEs and HCFs while ZnO had was less cytotoxic to HCFs versus HCLEs in comparison to CuO;	Zhou et al., 2014
TiO_2_ ENMs ZnO ENMs ZnO/PVP ENMs	19 ± 0.8 5 ± 0.32 6 ± 1.74	Continuous stirring with titanium tetraisopropoxide solution or zinc acetate dehydrate and then hydrolyzed by adding potassium hydroxide in ethanol	Light scattering spectroscopy; particle size, zeta potential, PDI	0.625–60 μL/mL	HCECs, ARPE-19 cells	24 h	MTT	ZnO/PVP NPs had a protective effect and the highest IC_50_ (24 μg/mL)	Agban et al., 2016
ZnO ENMs	15–50	Commercially purchased	Field emission scanning electron microscope	31.5–125.0 µmol/L	Murine photoreceptor cell line	6 or 24 h	Cytotoxic effect (LDH release assay, ROS, mitochondria membrane potential)	Induced cytotoxicity via potassium channel block and ATPase inhibition	Chen et al., 2017
ZnO ENMs	10–35	Provided by a company	SEM, Zeta-potential	0–125 μmol/L in DMEM	Murine photoreceptor cell line (661 W)	6 h	Cytochrome-c ELISA, flow cytometry for mitochondrial membrane potential and ROS, apoptosis/necrosis, proteomic analysis	Induced mitochondria-induced murine photoreceptor cell death (collapse the mitochondrial membrane potential, generate excessive ROS, etc.)	Wang et al., 2018
ZnO ENMs	20–90 nm	Commercially purchased	SEM, zeta potential	1–16 µg/mL	Human Tenon Fibroblasts	24, 48, and 72 h	MTT, CCK8	Dose-dependent cytotoxicity	Yin et al., 2019
								Moderate time dependent cytotoxicity	Wang et al., 2020
ZnO ENMs	60 nm	Commercially purchased	TEM	2.5–10 µg/m	Murine photoreceptor cells (661 W cell line)	72 h	RT-CES	Dose-dependent cytotoxicity	Guo et al., 2015
ZnS ENMs	50–200	Synthesized using the biomass of bacterium *Brevibacterium casei* incubated with 5 mM ZnSO_4_	UV-visible spectrophotometer, XRD, FTIR spectrum, TEM and DLS	10–1000 nM	Primary mouse RPE cells	24, 48 and 72 h	MTT, intracellular ROS measurement, Flow cytometric analysis for live/dead cell assay with PI, Western blots with Akt antibody	Cytotoxicity over 600 nM and enhancing Akt activity in a dose-dependent manner	Bose et al., 2016
ZnO ENMs Al_2_O_3_ ENMs Fe_2_O_3_ ENMs CeO_2_ ENMs CuO ENMs TiO_2_ ENMs V_2_O_5_ ENMs MgO ENMs WO_3_ ENMs	50 nm 30 nm 10 nm 10 and 30 nm 50 nm 25 and 100 nm 100 nm 20 nm 15 nm	Procured from Engineered Nanomaterials Coordination Core as part of NHIR consortium	DLS	0.05–250 µg/mL	Human telomerase reverse transcriptase-immortalized corneal epithelial cells	24 h	Calcein AM, MTT, Oris^TM^ migration assay	V_2_O_5_, WO_3_, and ZnO ENMs markedly decreased cell viability at 50 µg/mL or less. Al_2_O_3_ CeO_2_ (10 and 30 nm), CuO, Fe_2_O_3_ and MgO significantly impacted viability only at highest concentration tested. Migration was significantly reduced by Al_2_O_3_ CeO_2_ 10 nm, CuO, Fe_2_O_3_ at ≥50 µg/mL. V_2_O_2_ and ZnO reduced migration at ≥5 µg/mL.	Kim et al., 2020
MENMs	10 nm core	Commercially purchased, coated in house	TEM	0–4 OD	MSC	24 h	Propidium Iodide staining and flow cytometry	No decrease in cell viability or multipotency	Snider et al., 2018
MENMs	100	Commercially purchased (iron oxide core coated with dextran bioconjugated to streptavidin)	DNA tethered and lipid coating	≤400 million/µL	Adult dog and human RECs	24 and 48 h	Cytotoxicity morphological analysis; CM-H_2_DCFDA staining, transfection efficiency (fluorescence microscopy), ROS and necrosis (flow cytometry)	High transfection efficiencies without ROS formation or necrosis	Prow et al., 2006
MENMs	50 nm	Commercially purchased Covalently functionalized via EDC chemistry	UV-vis spectroscopy (thiocyanate assay)	0.001 µM–1 µM	HRECs	24 h	Dose-response analysis, MTT, cell migration with and without 80 ng/mL VEGF	No decrease in cell viability or migration due to MNPs	Amato et al., 2020
SPIO ENMs	50	Commercially purchased	TEM, Zeta potential	4–46 μg/mL					
SPIO ENMs	50	Commercially purchased	DLS, Zeta potential	1, 10 and 100 × 10^6^ SPIONPs/cells	Primary rabbit CECs	3 and 6 h	MTT, TEM, Homotypic adhesion assay, immunocytochemistry (ZO-1 and anti-Ki67), flow cytometry analysis (Ki67), measurement of corneal endothelial cell pump function	SPIONPs labelling of rabbit CECs does not affect cell functions at 16 μg/mL for 36 h	Bi et al., 2013
Fe_3_O_4_ ENMs MSIO nanofluid	7.2 ± 0.76	Synthesized using a modified high temperature thermal decomposition method	FTIR spectrometer, vibrating sample magnetometer	1–700 μg/mL	Primary bovine CECs	24, 48 and 72 h	MTT, live/dead cell assay, Cellular uptake after magnetic exposure	Significant differences in the metabolic activity of the CECs at 100 × 10^6^ SPIONPs/cell without cytoskeletal changes	Cornell et al., 2016
					Transformed rat RGC-5 cells	24 h	MTT, inductive-coupled plasma mass spectroscopy, induction of *HPSs 72*	No cytotoxicity up to 30 μg/mL with high cellular uptake up to a 52.5%. successful induction of *HPSs 72*.	Bae et al., 2016
**In vivo**									
CeO_2_ ENMs	Not specified	Not specified	Not specified	1 μL of 1 mM (172 ng)	Mutant mice with targeted deletion of the Vldlr gene (B6; 129S7- *Vldlr*^tm^ [1]^Her^/J; *Vldlr*^−/−^)	IVT; once (at P28); 7 days	Expression of cytokine genes (PCR array, Western blots), functional network analysis	Inhibited pro-inflammatory cytokines, pro-angiogenic growth factor and up- regulation of several cytokines and anti-angiogenic genes. CeO_2_ NPs inhibited the activation of ERK1/2, JNK, p38 MAP kinase, and Akt.	Kyosseva et al., 2013
CeO_2_ ENMs	18.2–50.7	Commercially purchased	FE-SEM, zeta potential and size distribution	65 and 85 mg/kg	Wister rats	PO; twice (one week before and after of STZ injection); 8 weeks	STZ induced diabetic rat model; antioxidant properties (measurement of lipid peroxidation), change (H&E), morphological	CeO_2_ NPs reduced oxidative stress and improved the histopathology and morphological abnormalities of dorsal root ganglion neurons	Najafi et al., 2017
CeCl_3_@mSiO_2_	87.6 ± 8.9	Mixed both mSiO_2_ NPs and CeCl_3_ power in acetone solution	TEM, DLS, spectrophotometer, UV-Vis	10 and 20 mg/kg	Wister rats	IP; twice a week; 8 weeks	STZ induced diabetic rat model; clinical exam, H&E, Biochemical analyses (MDA, GSH, SOD and GPx levels)	Antioxidant activity and antiglycation effect in the lens	Yang et al., 2017
CeO_2_ ENMs	20 nm	Ce(NO_3_)_3_ added to buffer with Sodium acetate and ethylic acid and stirred before dilution, heating, and 5 cycles of centrifugation and resuspension	TEM, XPS	80 µg/mL	Japanese white rabbit SD rat	1, 6, 12, 24 h 3, 7, 14 days	Slit lamp at each time point, modified Draize test, fluorescein staining 6 h post treatment alkali burn (0.9 M sodium hydroxide) antineovascularization and induced inflammation	No abnormal changes reported, fluorescein confirmed normal epithelium. Neovascularization decreased after treatment with CeNPs	Zheng et al., 2019
	10 and 100 nm	Purchased							
TiO_2_ ENMs (P25)	21	Commercial type	Spectrophotometer	1 mg/L	New Zealand White rabbits	Topical; once; 72 h	Acute eye irritation test (USEPA, 1998, and OECD405, 2002, guidelines)	Minimal irritation (conjunctival redness)	Warheit et al., 2007
TiO_2_ ENMs	<75	Commercially purchased	Not specified	0.5 mg/mL	Zebrafish embryos (*Danio rerio*, AB strain)	Exposed unit postfertilization; 72 h	Evaluation eye development and retina (IHC, whole mount in situ hybridization)	No embryonic development or retinal neurotoxicity	Wang et al., 2014
TiO_2_ ENMs	<75 nm	Commercially purchased	N/A	100 µg/mL	New Zealand white rabbits	Topical installation for 1 and 4 days	Ocular surface staining, phenol red thread test, tear sample, impression cytology, SEM	TiO_2_ treated groups had higher surface staining, no difference in tear secretion before and after exposure but LDH activity was 2-fold higher and MUC5AC conc was higher for 1 day	Eom et al., 2016
								treated rabbits. TiO_2_ treated eyes had lower PAS-positive conjunctival goblet cell density and the median (IQR) goblet cell area per unit Area for TiO_2_ group was lower than control	
TiO_2_ ENMs	42 nm	Commercially purchased	TEM, DLS, zeta-potential	0.25 and 0.5 ng per eye, 1 µL volume	C57BL/6 mice	Intravitreal, once	Retinal function, IOP, fundus photography, fundus fluorescein angiography, laser speckle flowgraphy, optical coherence tomography, electroretinogram	TiO_2_ diffuses from injection site and observed various injuries to retinal structure and function	Chan et al., 2021
ZnO ENMs	100	Commercially purchased	Not specified	500 mg/kg	Lewis rats	Topical; once	Dry eye model (scopolamine hydrobromide SC); Clinical scoring, phenol-red cotton thread test, tear evaluation (TUNEL, TNF- a, mucin)	The tear LDH level, TNUEL positive cells, TNF-a level and inflammatory cell infiltration on the ocular surface were higher in the dry eye model than the normal eyes	Han et al., 2017
ZnO ENMs	30	Commercially purchased	XRD, Fourier transform infrared spectroscopy	500 mg/kg	Sprague Dawley rats	Oral; once; 90 days	Histopathological changes with H&E stain	Retinal atrophy	Kim et al., 2014
ZnO ENMs V_2_O_5_ ENMs	50 nm 100 nm	Procured from Engineered Nanomaterials Coordination Core as part of NHIR consortium	DLS	50 µg/mL	New Zealand white rabbits	Topical six times daily final timepoint 105 h	Mechanical wound healing model	Corneal epithelial wound healing was significantly delayed by ZnO. Hyperspectral darkfield microscopy showed transcorneal penetration of ZnO and V_2_O_5_ in wounded and unwounded corneas.	Kim et al., 2020
Fe_3_O_4_ ferrofluid	10	Monodispersed Fe_3_O_4_ particles suspended in a fluorocarbon carrier oil	XRD	0.1 μM	Sprague Dawley rats	Oral; once; 90 days	Histopathological changes with H&E stain	Retinal atrophy	Park et al., 2014
MSIO nanofluid	7.2 ± 0.76	Synthesized using a modified high temperature thermal	FTIR spectrometer, vibrating sample magnetometer	30 μL/mL	Sprague Dawley rats	IVT and AC injection; once; 5 months	Functional and morphological changes (ERG, endothelial cell count, IOP, Histology and IHC)	No toxicity on the retina and no IOP changes	Raju et al., 2011
				5 mg/mL					
**Ex vivo, etc.**									
ZnO/PVP ENMs	6 ± 1.74	Continuous stirring with titanium tetraisopropoxide	Light scattering spectroscopy; particle size, zeta potential, PDI	ZnO/PVP:collagen ratios; 0.25, 0.5 and 1:1 *w*/*w*	Sprague Dawley rats New Zealand White rabbits	Intravitreal infusion for 30 min; once;	Diffusion behaviour, histology, TEM	Locally induce HSPs 72 in RGCs	Bae et al., 2016
		solution or zinc acetate dehydrate							
TiO_2_ ENMs	25	Mix titanium- diisopropoxide- bis(acetylacetonate) and 2-hydroxyethyl methacrylate (HEMA) under constant stirring	Powder diffractometer (XPD)	-	NP cross-linked collagen shields (for sustained delivery of pilocarpine hydrochloride)	14 days	Shield transparency, mechanical strength, swelling capacity and bioadhesive properties, release of zinc ions and PHCl	Collagen shields cross-linked with ZnO/PVP NPs released pilocarpine over 14 days offering a sustained release treatment option for glaucoma	Agban et al., 2016
MENMs	50 nm	Commercially purchased Covalently functionalized via EDC chemistry	UV-vis spectroscopy (thiocyanate assay)	0.001 µM–1 µM	C57BL/6J Mouse Retinal Explants	3 days	Biocompatibility and dose- response with drug-functionalized MNPs	MNPs did not induce apoptosis. MNPs loaded with octreotide showed increased bioactivity	Amato et al., 2020
					Hybrid materials of TiO_2_ NPs and poly-HEMA for IOL		In situ generated TiO_2_ NPs to enhance the refractive index of poly-HEMA hydrogels	TiO_2_ hydrogel were obtained flexible polymer lenses with high surface quality, shape memory and superior optical properties	Hampp et al., 2017

AC, anterior chamber; Akt, protein kinase B; APRE-19, human retinal pigment epithelial cell line; CEC, corneal epithelial cells; CeCl_3_, cerium(III) chloride; CeCl_3_@mSiO_2_, cerium(III) chloride loaded mesoporous silica; COMET, single-cell gel electrophoresis; DLS, dynamic light scattering; ELISA, enzyme-linked immunosorbent assay; ERG, electroretinogram; ERK, extracellular signal-regulated kinase; FE-SEM, field emission scanning electron microscopy; FTIR, Fourier transform infrared; GPx, glutathione peroxidase; GSH, glutathione; HCF, human corneal fibroblasts; HCLE, human corneal epithelial cells; HLE, human lens epithelial cells; *HPSs*, heat shock proteins; HR-SEM, high resolution scanning electron microscopy; H_2_O_2_, hydrogen peroxide; IHC, immunohistochemistry; ILSI, international life sciences institute; IOL, intraocular lens; IOP, intraocular pressure; IP, intraperitoneal injection; IVT, intravitreal injection; JNK, c-Jun N-terminal kinase; LDH, lactate dehydrogenase; MDA, malondialdehyde; MSIO, magnetically softened iron oxide; MTT, 3-(4,5-dimethylthiazol-2-yl)-2,5-diphenyltetrazolium bromide; OECD, organization for economic co-operation and development; PCO, posterior capsular opacification; PCR, polymerase chain reaction; PDI, polydispersity index; PI, propidium iodide; PO, per os (oral administration; PVP, polyvinylpyrrolidone; p38 MAP kinase; REC, retinal endothelial cells; RGC, retinal ganglion cells; ROS, reactive oxygen species; RPE, retinal pigment epithelium; SC, subcutaneous; SEM, scanning electron microscopy; SOD, superoxide dismutase; SPIONPs, superparamagnetic iron oxide nanoparticles; STZ, streptozotocin; TEM, transmission electron microscopy; TNF-a, tumor necrosis factor-a; TUNEL, terminal deoxynucleotidyl transferase dUTP nick end labelling; USEPA, united states environmental protection agency; XRD, X-ray diffraction; ZO-1, zonular occludens-1.

## Data Availability

Not applicable.
